# Is Type and Grade of Emphysema Important for Bone Mineral Density and Aortic Calcifications?

**DOI:** 10.3390/jcm13133947

**Published:** 2024-07-05

**Authors:** Danica Vuković, Danijela Budimir Mršić, Ivan Ordulj, Frano Šarić, Mirko Tandara, Kristian Jerković, Antonela Matana, Tade Tadić

**Affiliations:** 1 Clinical Department of Diagnostic and Interventional Radiology, University Hospital Split, Šoltanska 2, 21000 Split, Croatia; danica.vukovic333@gmail.com (D.V.); iordulj@gmail.com (I.O.); fsaric33@gmail.com (F.Š.); mirko.tandara@gmail.com (M.T.); jerkovickristian@gmail.com (K.J.); 2 School of Medicine, University of Split, Šoltanska 2, 21000 Split, Croatia; 3 University Department of Health Studies, University of Split, Ruđera Boškovića 35, 21000 Split, Croatia; antonela.matana@gmail.com

**Keywords:** emphysema, COPD, bone mineral density, calcifications, chest CT

## Abstract

**Background:** Chronic obstructive pulmonary disease has extrapulmonary manifestations, such as cardiovascular diseases and osteoporosis. The purpose of this research was to determine the relationship between the type and extent of emphysema with thoracic aorta calcification (TAC) and bone mineral density (BMD) at Th4, Th8, and L1 vertebrae. **Methods:** Emphysema was described by computed tomography parameters (both Fleischner classification and low attenuation value percentage, LAV%) and the clinical FEV_1_/FVC ratio (Tiffeneau–Pinelli index, TI, TI < 0.7; TI > 0.7). **Results:** Of 200 included patients (median age 64, 33% women), signs of clinical obstruction (TI) were observed in 104 patients, which had significantly lower BMD and more heavy TAC. BMD correlated negatively with LAV%, Rho = −0.16 to −0.23, while a positive correlation of aortic calcification with LAV% was observed, Rho = 0.30 to 0.33. Multiple linear regression showed that age and TI < 0.7 were independent predictors of BMD, β = −0.20 to −0.40, and β = −0.21 to −0.25; age and hypercholesterolemia were independent predictors of TCA, β = 0.61 and β = 0.19. **Conclusions:** Clinical TI and morphological LAV% parameters correlated with BMD and TAC, in contrast to Fleischer-graded emphysema, which showed no correlation. However, only TI was an independent predictor of BMD, while the morphologically described type and extent of emphysema could not independently predict any extrapulmonary manifestation.

## 1. Introduction

Pulmonary emphysema is an abnormal and permanent enlargement of the airspaces beyond the terminal bronchioles, along with the destruction of the alveolar wall, without significant fibrosis [1,2]. It is the most common type of chronic obstructive pulmonary disease (COPD), a heterogeneous and chronic condition characterized by irreversible airflow limitation, caused by overstated pulmonary inflammatory response, activated by exposure to noxious gases (smoking, traffic gasses, etc.) and particles [3,4,5]. It is characterized by chronic inflammatory processes in the lung parenchyma which promotes structural remodeling and damage to the small and large airways, impairing the elastic recoil of the lung, and ultimately reducing lung function [6]. Early diagnosis of emphysema is made possible through the use of computed tomography (CT) [7,8]. Additionally, CT enables the accurate measurement of the extent of emphysema in the lungs, which is crucial for determining the severity of the condition [7]. Moreover, it enabled the in vivo detection of emphysematous changes in lung parenchyma in patients, with observations indicating its occurrence in individuals without airflow restrictions and without clinically manifested obstruction, i.e., COPD [9]. The diagnosis of COPD requires the spirometric demonstration of persistent airflow limitation, as defined by a post-bronchodilator reduction in the ratio of forced expiratory volume in one second (FEV_1_) to forced vital capacity (FVC) below 70% in patients with appropriate symptoms and history [3]. Spirometry is not only used for diagnosis but also for quantifying the degree of obstruction and then further categorizing it according to the Global Initiative for Chronic Obstructive Pulmonary Disease (GOLD) standards [3,4,8].

Despite being primarily a pulmonary disease, COPD also presents significant extrapulmonary manifestations and complications [6,10], which are incorporated into the definition and management of COPD as outlined in the GOLD guidelines [11,12]. Not only does COPD have extrapulmonary manifestations but it also coexists with other conditions known as comorbidities [10]. Some of the extrapulmonary complications/comorbidities include increased arterial stiffness, systemic hypertension, osteoporosis, skeletal muscle atrophy, and others including cancer [10,13,14,15]. The exact cause-and-effect relationship is still not clearly established, so there are several possible explanations. Research indicates that pro-inflammatory agents and reactive oxygen species (ROS) may spread beyond the lungs into the bloodstream, leading to extrapulmonary complications. On the other hand, some believe it is all a result of shared risk factors including advanced age, physical inactivity, bad-quality diet, cigarette smoking, hypoxia, and systemic inflammation. 

One of the most important comorbidities is cardiovascular disease (CVD) since it has been stated that CVD is the main cause leading to morbidity and mortality associated with COPD, with approximately 30% of all COPD patients dying as a result of cardiovascular manifestations [6]. Not only persistent pulmonary inflammation (disease itself) but also increased oxidative stress resulting from corticosteroids (used for disease management) are thought to be the main factors contributing to atherosclerosis leading to CVD in individuals with COPD [16,17,18]. The most common CVDs are ischemic heart disease (IHD), arrhythmias, and heart failure. A study conducted by Williams et al. demonstrated that coronary artery calcifications (indicating coronary artery disease, the main cause of IHD) are increased in patients with COPD and are associated with increased morbidity and mortality. Atherosclerosis affects not only the coronary arteries but also the aorta and smaller peripheral blood vessels [6]. On the CT scans, the extent of arterial atherosclerotic calcification, a predictor of overall cardiovascular risks, can be visualized and measured [17,19].

Furthermore, chronic systemic inflammation is also associated with reduced bone mineral density (BMD) in these patients, potentially leading to osteoporosis [20,21]. The causal link between COPD and osteoporosis is not yet well defined, but it potentially includes smoking effects on bone tissue, inactivity, nutritional deficits, and glucocorticoid use as well as chronic inflammation [19,20]. Patients with osteoporosis are at an increased risk of developing fractures, leading to significant morbidity and mortality, particularly patients with COPD where vertebral fractures can further compromise already reduced pulmonary function [21,22]. Fractures also lead to more frequent hospitalizations and decreased exercise capacity and overall mobility increasing the probability of infection and possible exacerbation which all lead to poor quality of life [23].

Detection of emphysema is possible by its direct visualization on the chest CT scans. Recently, a classification from the Fleischner Society divided emphysema into three types: centrilobular (subclassified as trace, mild, moderate, confluent, and advanced destructive emphysema), panlobular, and paraseptal (subclassified as mild or substantial), including some additional visual features [24]. Also, on CT scans, it is possible to determine the extent of emphysema using threshold-based identification of low attenuation volume percentage (LAV%) in order to precisely quantify the exact percent of emphysematous parenchyma [7,19,25]. Apart from the mentioned morphological CT emphysema presentation and determination of its direct extension, one clinical parameter, the spirometric ratio between the FEV_1_ and FVC, called the Tiffeneau–Pinelli index (TI), is used in the diagnosis of COPD. A ratio below 0.7, even post-bronchodilator, is indicative of COPD [3,4].

Given that there are several different diagnostic approaches to emphysema, the purpose of our research was to determine the relationship between the type and the extent of emphysema using both the morphological chest CT (Fleischner classification and LAV%) parameters and a clinical (TI) parameter with common extrapulmonary manifestations, such as calcifications of the thoracic aorta (TAC) and spine BMD, both extracted from the same CT chest scans. To our knowledge, we did not find a study that linked both the type and extent of emphysema with TACs and spine BMD in a cohort of patients with emphysema, in which only part of the study population had COPD. We hypothesized that the patients with a larger volume of lung parenchyma affected by emphysema and those with a destructive subtype of emphysema would have lower BMD and would have more severe calcifications of the thoracic aorta. We believe that by obtaining as much information as possible from one single examination, we can detect and treat chronic diseases in time and thus reduce the burden on the medical system.

## 2. Materials and Methods

### 2.1. Study Design and Participants

**Figure 1:**
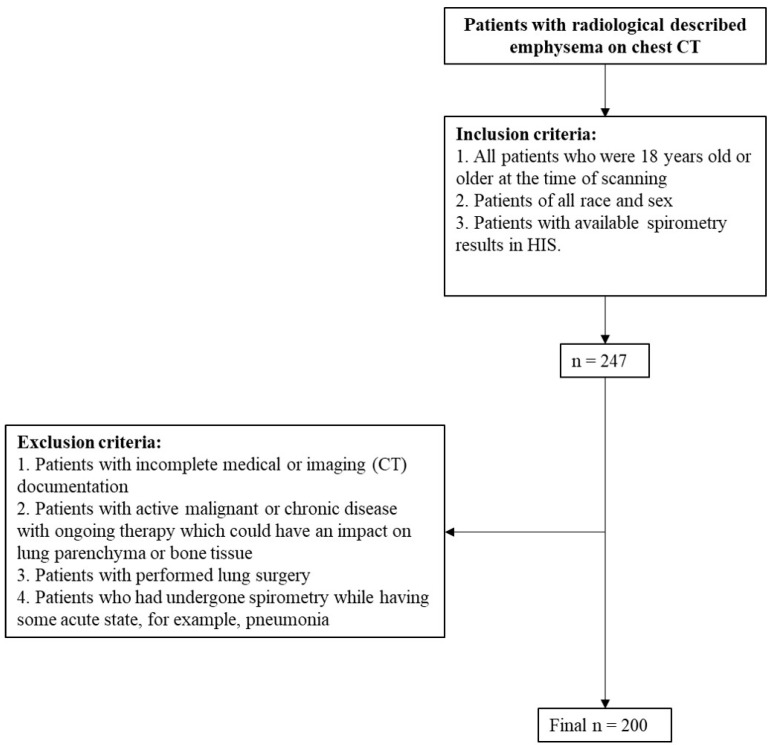
Flow chart of included study population.

This retrospective cross-sectional study was conducted at a tertiary radiology department of an academic medical center between January and February of 2024. In the given time period, we retrospectively collected and analyzed patients who have undergone chest CT scans between 20 May 2022 and 1 January 2024 and have had radiologically described changes in the lung parenchyma characterized as emphysema. The patient’s medical history was retrieved from the Hospital Information System (HIS). The inclusion criteria were as follows: (1) all patients who were 18 years old or older at the time of scanning; (2) patients of all races and all sexes; and (3) patients with available spirometry results in the HIS. The exclusion criteria were as follows: (1) patients with incomplete medical or imaging (CT) documentation; (2) patients with active malignant or chronic disease with ongoing therapy which could have an impact on lung parenchyma or bone tissue; (3) patients with performed lung surgery; and (4) patients who had undergone spirometry while having some acute state, for example, pneumonia. All is shown in detail in Figure 1.

All patients performed spirometry no more than six months prior to chest CT acquisition. From the spirometry findings, we looked at the value of the TI. We initially divided the patients according to the TI value after bronchodilator application, assuming that patients with a TI value below 0.7 have clinical symptoms and signs of obstruction. From the initial 247 patients who met the inclusion criteria, after further filtering by the mentioned exclusion criteria (*n* = 47 patients), a total of 200 Caucasian European patients were included in the analysis.

### 2.2. Analysis of Imaging Data

Lung scanning was performed using the 128-slice CT Siemens Somatom Definition AS, Germany. All patients were requested to hold their breath at maximal inspiration in the supine position. CT parameters used were a tube current of 113 to 200 mAs and a tube voltage of 120 kVp, automatically adjusted depending on a patient’s physical characteristics. Non-contrast scans and scans in the venous phase (approximately 60 s delay) were performed with lung parenchyma reconstructions from non-contrast scans.

#### 2.2.1. Lung Parenchyma Analysis

**Figure 2:**
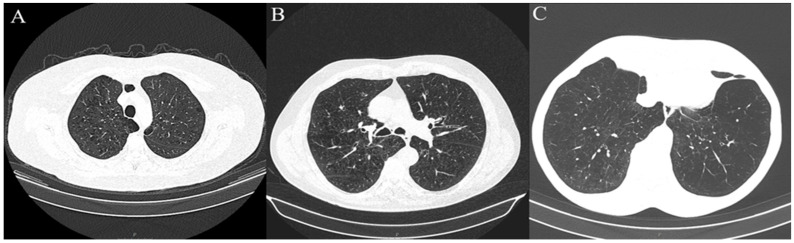
Axial chest CT scans of included patients demonstrating different emphysema types according to Fleischner guidelines. (**A**) Moderate centrilobular emphysema located in upper lung zone of lung parenchyma. (**B**) Confluent centrilobular emphysema located in middle lung zone of lung parenchyma. (**C**) Advanced destructive centrilobular emphysema located in lower lung zone of lung parenchyma. Patients (**B**,**C**) also have mild pareseptal and substantial paraseptal emphysema, respectively.

**Figure 3:**
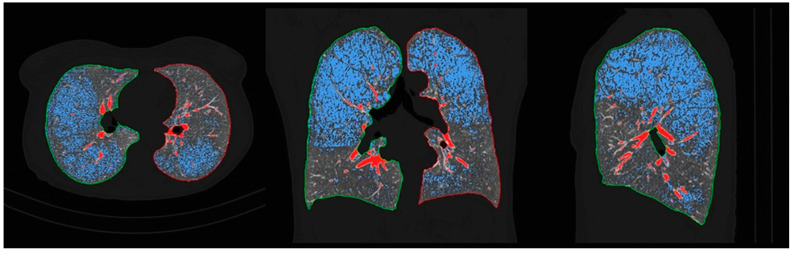
Axial, coronal, and sagittal chest CT scan with postprocessing in Syngo.Pulmo3D. Blue zones represent low attenuation areas/volumes (LAV%) indicating emphysema.

Lung CT imaging data were viewed and analyzed on a dedicated workstation with Syngo.via and Syngo.Pulmo3D (Siemens Healthcare, Erlangen, Germany) on lung and mediastinal window settings (lung: WW 1500 HU, WL –600 HU, mediastinal WW 400 HU, WL 40 HU). The lung scans were separately reviewed by two experienced radiologists with more than twenty and ten years of expertise in the field of thoracic radiology, in a random order, and blinded to the original scan report using the Fleischner Society guidelines. Each reader analyzed and recorded the type of emphysema as follows: centrilobular (trace, mild, moderate, and advanced destructive), paraseptal (mild and substantial), and panlobular emphysema (Figure 2). If more than one type of emphysema was present, they noted each one individually. As for the extent of the involvement of the lung parenchyma by emphysema, LAV% was measured automatically for each lung individually as well as the total value for both lungs (Figure 3) on Syngo.via postprocessing VB60A_HF08 software. Interobserver variability for the type of emphysema was high; Kappa = 0.866, *p* < 0.001.

#### 2.2.2. Vascular Calcification Measurement

**Figure 4:**
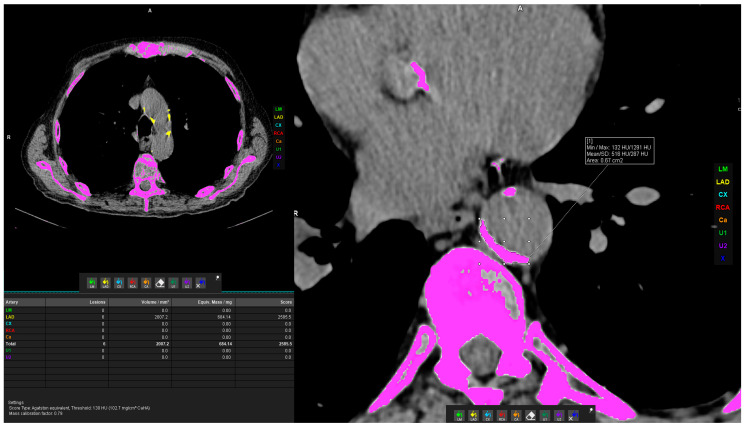
Axial chest CT scans of calcification measurement. (**Left**) Thoracic aorta calcification (TAC) measurement using postprocessing software VB60_HF08. (**Right**) Freehand ROI measurement of calcification volume.

For the volume measurement of TAC, the Siemens Syngo.via postprocessing VB60A_HF08 software was used with the “Ca Scoring” tool on unenhanced CT scans. The tool semiautomatically estimates calcifications on CT scans and can calculate the Agatston score. If the measurement was not automatically possible, in case vascular calcifications were too close to the bone structures (e.g., vertebral osteophytes) or were extending to adjacent arteries, the freehand region of interest (ROI) was used to delineate calcifications of interest and calculate the calcification volume. Measurements were performed by three general radiologists who underwent extensive training prior to the measurements described above. The measurement procedure is shown in Figure 4.

#### 2.2.3. Bone Density Measurement

**Figure 5:**
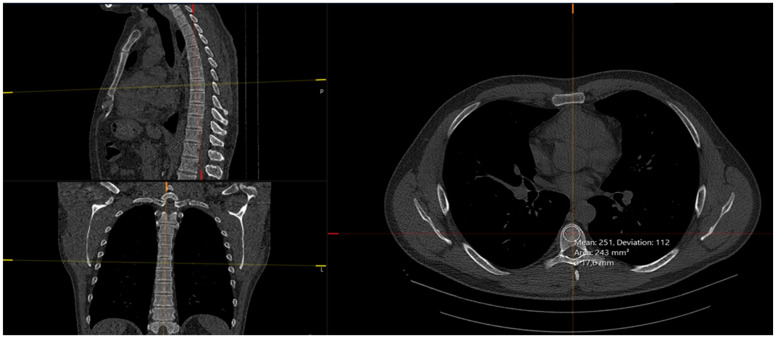
Axial, coronal, and sagittal chest CT scan in bone window representing measurement procedure of bone density with all planes placed perpendicular to measured vertebral body and ROI positioned in upper part of vertebral body.

The CT number of each voxel, known as HU, is associated with tissue density. The ROI was placed in axial view in the upper part of the vertebral body between the endplate and the entrance of the vessels at the anterior midportion, in a homogenous area of trabecular bone in the vertebral bodies of Th4, Th8, and L1 vertebra (Figure 5). ROIs were as large as possible, and focal heterogeneous areas were avoided (bone island, bone cyst, deep Schmorl’s nodes, hemangiomas). If any of the vertebral bodies were fractured or altered in any other way and if the L1 vertebra was not visualized in the scanning field, bone density was measured in a visible adjacent normal vertebra. The measurements were performed by two trained radiologists specialized in the field of thoracic radiology, who had special education prior to the measurement procedures described above. Interobserver coefficients were as follows: for Th4, ICC = 0.998, *p* < 0.001; for Th 8, ICC = 0.998, *p* < 0.001; and for L1, ICC = 0.994, *p* < 0.001.

#### 2.2.4. Statistical Analysis

The normality of the distribution of continuous variables was tested by a one-sample Kolmogorov–Smirnov test. Continuous variables with normal distribution were reported as mean ± standard deviation (SD) while non-normal variables were presented as median (interquartile range (IQR)). Categorical variables are presented with frequencies (percentages). Differences in categorical variables were analyzed by using a chi-square test, while the Student’s *t*-test and Mann–Whitney test were used to determine differences between two groups according to the distribution of the continuous variable. Correlations between variables were tested by applying Spearman’s rank correlation test. Finally, we performed multivariate linear regression analyses in order to assess the association of bone density at the observed three levels (Th4, Th8, L1) with the predictors that were significant in univariate models. We also performed a multivariate linear regression analysis in which a log transformation of calcification volumes of thoracic aorta was a dependent variable, and predictors significant in univariate models were independent variables. p values of less than 0.05 were considered statistically significant. Statistical analysis was carried out using Statistical Package Software for Social Science, version 28 (SPSS Inc., Chicago, IL, USA).

## 3. Results

A total number of 200 patients were included in the study, including 134 men (67%) and 66 women (33%). The median patient age was 64 years (IQR, 12 years). More than three-quarters of the patients were smokers, and more than half were active smokers. The most common comorbidity was arterial hypertension (43.5%), followed by hypercholesterolemia (23.5%), Table 1. No significant difference was observed in TACs between men and women (*p* = 0.834, Mann–Whitney test). Contrary, more TACs were present in people with hypertension than in those without (*p* < 0.001, Mann–Whitney test). Borderline significance (*p* = 0.099, Mann–Whitney test) was detected for the association between TACs and hypercholesterolemia.

Analysis of CT scans detected different grades of centrilobular emphysema in 135 patients (67.5%), while paraseptal emphysema was described in 120 of them (60%). Panlobular emphysema was not detected. The median LAV% was 4.3 (IQR, 12.4). The median volume of CTA was 3592 mm^3^ (IQR, 18620 mm^3^). Bone mineral density was the highest at the Th4 level with a median of 166 HU (IQR, 74.5 HU) and lowest at the L1 level with a median of 122.09 HU (IQR, 38.7 HU), (Table 1).

Since the criterion for the diagnosis of COPD is postbronchodilator TI < 0.7, our study population was divided according to its value and presented comprehensively in Table 1. The number of patients, after dividing by the presence of clinical obstruction (TI < 0.7), was almost the same in both groups (104 vs. 96 patients). Patients with a TI < 0.7 were significantly older and had a clinically recorded exacerbation of the disease in the last year. Radiologically, advanced destructive emphysema was the most often described in these patients (*p* < 0.012), as well as a statistically significantly higher LAV% (Table 1).

Lower bone mineral density of vertebral bodies of Th4, Th8, and L1 was observed in patients with TI < 0.7 compared to TI ≥ 0.7 (*p* = 0.001, *p* < 0.001, *p* < 0.001, respectively). Also, more CTA was observed in patients with TI < 0.7 compared to TI ≥ 0.7 after the bronchodilator (*p* = 0.001), as shown in Table 2.

Similarly, lower bone mineral density expressed in HU was detected in observed vertebral bodies of Th4, Th8, and L1 in individuals with a higher total LAV%, left lung LAV%, and right lung LAV%; the correlation coefficients ranged from −0.163 to −0.235, *p* < 0.05 (Table 3). A low positive correlation of thoracic aorta calcifications with total LAV%, left lung LAV%, and right lung LAV% was observed; the correlation coefficients ranged from 0.302 to 0.33, *p* < 0.001 (Table 3). No significant correlation between emphysema type and bone density or aortic calcification was observed.

Multiple linear regressions using bone density at the observed three levels (Th4, Th8, L1) as dependent variables showed that of all significant univariate predictors, only age and TI < 0.7 were independent predictors of bone density at all three levels, with beta ranging from −0.207 to −0.406 (*p* < 0.004) for age and from −0.212 to −0.257 (*p* < 0.004) for TI < 0.7, as shown in Table 4.

Similarly, the multiple linear regressions using calcification volume of the thoracic aorta as the dependent variable showed that of all significant univariate predictors, only age and hypercholesterolemia remained independent predictors of volume TAC, beta 0.612, *p* < 0.001 for age, and beta 0.193, *p* = 0.001 for hypercholesterolemia, Table 5.

## 4. Discussion

The results of the study showed that a larger volume of lung parenchyma affected by emphysema, presented by a higher LAV% value, was negatively associated with vertebral BMD and positively with TACs in univariate analysis, compared to Fleischner’s type and grade of emphysema that showed no association. However, in multivariate analysis, this effect of LAV% was not confirmed to be independent. In other words, we found a clinical parameter, TI, to be more strongly associated than morphological CT parameters with the BMD, while emphysema itself, measured by both CT and clinical parameters, showed no independent effect on TAC. Moreover, age and hypercholesterolemia turned out to be the only independent predictors of volume TAC.

The studies investigating pathophysiological processes using both clinical and morphological parameters, such as CT, are rare. For a comparison of our results, regarding BMD, we used studies that also measured BMD on the CT scans. One study investigating the relationship between emphysema, calcification of the coronary arteries (CAC), and TACs and BMD, on a low-dose chest CT, in COPD patients did not find a correlation between the observed parameters [19]. They measured the average BMD of three vertebral bodies (T4, T7, and T10) in Hounsfield units. In contrast, Ohara and colleagues demonstrated that the value of BMD significantly correlated with the quantity of lung parenchyma affected by emphysema [25]. Bone attenuation was measured at the level of the T4, T7, T10, and L1 vertebra and each measurement was negatively correlated with the extent of emphysema. By performing multiple regression analysis, this study even pointed out that the percentage of lung low attenuation area (LAA%), equivalent to LAV%, could predict BMD in male COPD patients without ongoing corticosteroid therapy. Furthermore, Jaramillo and al. showed that COPD, especially emphysema, was associated with low BMD after adjusting for the history of corticosteroid use, patient age, the amount of overall smoking, present smoking, and COPD exacerbations [26]. Mean BMD was calculated from the minimum of three vertebral bodies from T6 to L1. It is known that the main determinant of BMD is age, but sex, obesity, and many other conditions can affect bone turnover [27]. Following the above, a potential difference in these results might be explained by the difference within the study population, that is, what was the percentage of elderly patients and the percentage of women in whom lower average BMD values can be expected in the total study population. Furthermore, some studies included only COPD patients while we included all patients who had radiologically described emphysema, i.e., not all patients had a diagnosis or criteria for a diagnosis of COPD (TI < 0.7). Not only COPD diagnosis but also the severity of the disease, expressed in the GOLD stage, was shown to have an impact on the average BMD by some studies, meaning patients with a higher GOLD stage tend to have lower BMD [19,28]. Therefore, the percentage of individual GOLD stages in the studied population could have an impact on BMD values. Also, the potential discrepancy between study results might be due to the share of active smokers in the total examined population because smoking is recognized as an independent factor in the development of osteoporosis [29]. Finally, the location of the BMD measurement itself might be the cause of the conflicting findings. Several studies have shown that the average value of BMD gradually decreases craniocaudally; therefore, the location of the BMD measurement itself might have had an impact on the result [14,27].

In the case of aortic calcifications, a study by Dransfieal et al. showed that the percentage of LAA% remained a significant predictor of the calcium score of the thoracic aorta after the adjustment for the other covariables [30]. Today, COPD is not, according to recent studies, considered an isolated lung disease, but it is considered only a pulmonary manifestation of systemic endothelial dysfunction where there is a whole spectrum of inflammatory processes that take place simultaneously in multiple organs causing the so-called multimorbidity without a clear indication of which disease came first [31]. Our study confirmed the percentage of LAV% was more strongly associated than Fleischner’s visual type of emphysema with TACs. Perhaps the reason lies in the fact that we did not have a sufficient number of patients with each type of emphysema. Also, the calcification of atherosclerotic plaques is not necessarily present and detectable by a CT, especially in the early development of atherosclerosis. It has been described that patients with TACs have a higher incidence of cardiovascular mortality independent of other risk factors [30]. In our study, we also demonstrated that hypercholesterolemia was an independent predictor of TAC, which is a well-known risk factor for developing atherosclerosis. Emerging studies have shown hypertriglyceridemia to be a significant contributor to the development and progression of atherosclerosis and that lowering their serum levels could reduce the risk for CVD as well [32]. We suggest that future studies include this variable in the analysis to deepen the understanding of the underlying mechanisms. The process of atherosclerosis itself is accompanied by inflammation that weakens the artery wall and can lead to narrowing of the lumen or aneurysmal expansions that can result in acute aortic syndromes, potentially life-threatening conditions including aortic dissection, intramural hematoma, or penetrating aortic ulcer [31,33]. Nonetheless, Romme et al. demonstrated that CAC and TAC predict all-cause mortality after adjustment for age, sex, FEV_1_, and pack-years of smoking [19]. Many studies have shown that a COPD patient is just as likely to die from cardiovascular disease as from respiratory disease [6,10,31]. In addition, the emphysematous type of COPD, in which loss of elastic recoil occurs due to the destruction of alveoli, is considered a potential contribution to the development of pulmonary artery hypertension and right ventricular dysfunction, thus increasing the possibility of developing CVD [31]. Therefore, we consider it important to describe these changes in arterial walls along with the analysis of the lung parenchyma in these patients.

Finally, apart from CT parameters for emphysema, our study demonstrated that a clinical parameter, postbronchodilator TI < 0.7, was the most strongly associated with observed extrapulmonary manifestations, especially with the BMD, since it independently predicted BMD of vertebral bodies. We showed that patients with TI < 0.7 had a higher prevalence of advanced destructive centrilobular emphysema, a larger LAV%, and a lower BMD of the measured vertebral bodies, as well as a larger volume of TAC. This was to be expected since various studies have shown that centrilobular emphysema affects the small airways, causing airflow limitation and air-trapping clinically presented as obstruction [5,6], contrary to paraseptal emphysema in which small airways are relatively not affected [34]. As for the CT studies, the results including clinical parameters, such as TI, are conflicting; some studies have shown a strong association between COPD and low BMD [8,9], while some have not [35,36]. The results vary significantly depending on the study population, as discussed earlier.

Although it was not the subject of current research, we think it is important to mention sarcopenia, another important extrapulmonary complication of COPD, which is a major factor in the assessment of the patient’s overall physical health. Studies have proven that COPD individuals with lower muscle mass had worse pulmonary function tests and had an increased risk for developing osteoporosis, whose potentially harmful effects we elaborated on previously [37]. Not only does the skeletal muscle dysfunction affect respiratory muscles (intercostal muscles, diaphragm), but it also affects limb muscles and thus leads to decreased exercise and functional capacity in COPD patients [38]. Furthermore, skeletal muscle dysfunction affects around 20% of COPD patients and is associated with higher morbidity and mortality [39].

With the advent of artificial intelligence (AI) technology, machine learning and deep learning models have applications in osteoporosis and vascular calcification detection but also in attempts at COPD diagnosis. Although, until now, the diagnosis of COPD was made based on clinical parameters and imaging methods, new studies have shown that AI-based quantification of emphysema correlates well with TI [40]. Furthermore, radiological parameters for emphysema detection and classification were found to be significantly related to COPD severity but also could effectively distinguish between patients with severe and moderate COPD [41]. As we mentioned earlier, TACs can be an indicator of the development of CVD, a major cause of morbidity and mortality. As a result, scientists have developed AI models that assist radiologists in the rapid detection and quantification of vascular calcifications thus providing important information regarding potential cardiovascular risk [42]. One study showed that the AI model detected TACs with a sensitivity of 98.4% [43], which would make the diagnostic process significantly easier and faster for the radiologist. As for BMD, recent studies have demonstrated the promising potential of using AI tools for CT to opportunistically predict and classify osteoporosis without the need for performing additional studies, such as densitometry [44]. Although AI significantly helps in the detection and diagnosis of various diseases, we believe that the radiologist is nevertheless crucial in the critical assessment of the obtained data and their holistic integration into a quality assessment of the patient.

The main strength of our study is that we are, as far as we know, after searching the available literature, the only study that has linked two morphological parameters of emphysema seen on CT (visual type and quantitative extent) and one clinical parameter (clinical sign of obstruction) in order to investigate the associations of emphysema and its clinical manifestations with bone density and aortic calcifications on the same chest CT scans. That is, we studied the influence of the type of emphysema (according to the Fleischner Society classification), the quantitative amount of involvement of the lung parenchyma by emphysema (LAV%), and clinical signs of obstruction (TI < 0.7; ≥0.7) with the mentioned variables above. Although we only had 200 patients in the study, we believe that we avoided potential missing data due to strict exclusion criteria and thus strengthened the results of our retrospective study. However, despite strict exclusion criteria, the main limitation of our study is the fact that we retrospectively collected data and certain data were not available, for example, patients’ height and weight, body mass index (BMI), and systolic blood pressure which all could contribute to strengthening our study with more clinical details. Spirometry significantly depends on the motivation of the patient in the moment and the performance itself, which we did not attend, and we must take the results of “clinical obstruction” with caution. Some patients could underperform with false positive results for clinical obstruction and thus it could contribute to the results. It would be more accurate if the patients had performed it immediately after the CT scan was completed and thus the degree of the obstruction (GOLD stage) could be calculated from the FEV_1_ predicted value and further correlated with observed parameters. The CT scans were all performed in inspiration, although an expiration examination would have given us information on air trapping as a sign of small airway disease associated with emphysema. There was a relatively small number of patients in some of the subgroups of emphysema by Fleischner (panlobular emphysema was not detected). Future research could link the degree of clinical obstruction, i.e., the value of FEV_1_ predicted (GOLD category of COPD) with the mentioned parameters. Furthermore, future studies could focus on longitudinal assessments to track the progression of BMD and TAC changes with emphysema severity. Additionally, exploring the impact of specific emphysema subtypes in larger cohorts could yield valuable insights.

Even though COPD is considered a disease with a predominant pulmonary manifestation, further research is needed for a better understanding of the cause-and-effect relationship between the disease itself and synchronous systemic conditions (such as osteoporosis, atherosclerosis, or sarcopenia). It is necessary to clarify whether it is systemic inflammation and its consequent extrapulmonary manifestations or comorbidities due to shared risk factors. Although we did not answer this question, we did demonstrate that in our cohort there is a correlation between the mentioned parameters. So, following the results discussed above, we believe that reporting “radiological” indicators of cardiovascular risk and osteoporosis risk, in standard chest CT, can help in the early diagnosis and therapy of these patients. Therefore, we suggest that when assessing emphysema of the lung parenchyma, information on vertebral BMD and TACs should also be included in radiological reports in the future with the assistance of AI models.

## 5. Conclusions

To conclude, although one clinical and one morphological CT parameter (TI and LAV%, respectively) had significant effects in univariate analyses, only TI showed an independent prediction of bone density, while none of the parameters had an independent effect on aortic calcification in a cohort of emphysematous patients.

## Figures and Tables

**Table 1:** The demographic, clinical, and imaging characteristics of emphysematous patients in a total population sample, and in two groups of low and high TI.

		Total Sample	TI ≥ 0.7 (*n* = 96)	TI < 0.7 (*n* = 104)	*p* Value
Demographic Parameters and Habits	Gender				0.229 *
	Male	134 (67%)	60 (62.5%)	74 (71.2%)	
	Female	66 (33%)	36 (37.5%)	30 (28.8%)	
	Age; years, median (IQR)	64 (12)	62 (12)	67 (11.5)	0.001 ^#^
	Smoking				0.277 *
	Active	117 (58.5%)	61 (65.6%)	56 (54.4%)	
	Former	67 (33.5%)	27 (29%)	40 (38.8%)	
	Non-smoker	12 (6%)	5 (5.4%)	7 (6.8%)	
	Missing data	4 (2%)			
	Cigarette number/day	20 (10)	20 (10)	20 (20)	0.256 ^#^
Clinical Parameters	Arterial hypertension	87 (43.5%)	39 (40.6%)	48 (46.2%)	0.446 *
	Diabetes	23 (11.5%)	13 (13.5%)	10 (9.6%)	0.388 *
	Hypercholesterolemia	47 (23.5%)	26 (27.1%)	21 (20.2%)	0.5 *
	Exacerbations	40 (20%)	12 (12.4%)	28 (27.2%)	0.013 *
	Oral corticosteroids	42 (21%)	17 (17.5%)	25 (24.3%)	0.298 *
	Anti-osteoporotic therapy	14 (7%)	8 (8.2%)	6 (5.8%)	0.585 *
Radiological Parameters	**Emphysema subtype**				
	Trace centrilobular	32 (16%)	22 (22.9%)	10 (9.6%)	0.012 *
	Mild centrilobular	18 (9%)	10 (10.4%)	8 (7.7%)	0.623 *
	Moderate centrilobular	22 (11%)	9 (9.4%)	13 (12.5%)	0.507 *
	Confluent centrilobular	63 (31.5%)	29 (30.2%)	34 (32.7%)	0.762 *
	Advanced destructive centrilobular	45 (22.5%)	11 (11.5%)	34 (32.7%)	<0.001 *
	Mild paraseptal	49 (24.5%)	27 (28.1%)	22 (21.2%)	0.324 *
	Substantial paraseptal	71 (35.5%)	30 (31.3%)	41 (39.4%)	0.240 *
	Panlobular	0	0	0	-
	Total LAV%, median (IQR)	4.3 (12.4)	2.1 (3.7)	12.4 (19.4)	<0.001 ^#^
	Left lung LAV%, median (IQR)	4.2 (12.25)	1.8 (3.4)	12.5 (17.7)	<0.001 ^#^
	Right lung LAV%, median (IQR)	3.9 (13)	2.1 (3.1)	12.05 (19.6)	<0.001 ^#^

* Chi-square test, ^#^ Mann–Whitney test.

**Table 2:** Bone density (Th4, Th8 and L1) and thoracic aorta calcifications of emphysematous patients in a total population sample, and in two groups of low and high TI.

	TI ≥ 0.7	TI < 0.7	* p * Value
Bone density of Th4 (HU), mean ± SD	189.9 ± 48.8	165.7 ± 46.38	0.001 *
Bone density of Th8 (HU), mean ± SD	157.0 ± 45.8	130.6 ± 36.2	<0.001 *
Bone density of L1 (HU), mean ± SD	125.4 ± 37.9	99.8 ± 31.8	<0.001 *
Thoracic aorta calcifications (mm^3^), median (IQR)	410 (1648.3)	1531 (3219.2)	0.001 ^#^

* Student’s *t*-test, ^#^ Mann–Whitney.

**Table 3:** Spearman correlation coefficients (Rho) between total and individual lung LAV % and vertebral bone density and thoracic aorta calcifications.

	Total LAV%	Left Lung LAV%	Right Lung LAV%
Bone density of Th4 (HU)	Rho = −0.18, *p* = 0.012	Rho = −0.162, *p* = 0.024	Rho = −0.182, *p* = 0.011
Bone density of Th8 (HU)	Rho = −0.204, *p* = 0.004	Rho = −0.207, *p* = 0.004	Rho = −0.189, *p* = 0.008
Bone density of L1 (HU)	Rho = −0.219, *p* = 0.002	Rho = −0.226, *p* = 0.002	Rho = −0.193, *p* = 0.007
Thoracic aorta calcifications (vol./mm^3^)	Rho = 0.325, *p* < 0.001	Rho = 0.333, *p* < 0.001	Rho = 0.302, *p* < 0.001

**Table 4:** Multiple linear regression analysis showing independent predictors of bone density of Th4, Th8, and L1 (HU).

Bone Density of Th4 (HU)	Standard β	T	*p* Value
Age	−0.207	−2.907	0.004
Total LAV%	−1.725	−1.047	0.297
Left lung LAV%	1.022	1.182	0.239
Right lung LAV%	0.733	0.856	0.393
TI < 0.7	−0.212	−2.669	0.008
	R2 = 0.119		
**Bone density of Th8 (HU)**	**Standard β**	**T**	***p* Value**
Age	−0.359	−5.273	0.000
Total LAV%	−0.448	−0.284	0.776
Left lung LAV%	0.245	0.297	0.767
Right lung LAV%	0.219	0.268	0.789
TI < 0.7	−0.221	−2.936	0.004
	R2 = 0.195		
**Bone density of L1 (HU)**	**Standard β**	**T**	***p* Value**
Age	−0.406	−6.231	0.000
Total LAV%	−1.871	−1.241	0.216
Left lung LAV%	0.933	1.180	0.239
Right lung LAV%	1.013	1.294	0.197
TI < 0.7	−0.257	−3.565	0.000
	R2 = 0.263		

**Table 5:** Multiple linear regression analysis showing independent predictors of calcification volumes of thoracic aorta.

Volume of Thoracic Aorta Calcifications	Standard β	T	*p* Value
Age	0.612	10.715	<0.001
Total LAV%	0.900	0.729	0.467
Left lung LAV%	−0.341	−0.524	0.601
Right lung LAV%	−0.500	−0.781	0.436
TI < 0.7	0.089	1.467	0.144
Hypercholesterolemia	0.193	3.502	0.001
Arterial hypertension	0.060	1.051	0.295
	R2 = 0.539		

## Data Availability

The datasets are available upon reasonable request to the corresponding author.
